# Should all patients with aortic aneurysm and bicuspid aortic valve also undergo hemiarch?

**DOI:** 10.1016/j.xjon.2020.12.021

**Published:** 2021-02-16

**Authors:** Isao Anzai, Jacob Kriegel, Ilya Kim, Christian Pearsall, Matthew Lewis, Marlon Rosenbaum, Giovanni Ferrari, Isaac George, Hiroo Takayama

**Affiliations:** a Division of Cardiac, Thoracic, and Vascular Surgery, Department of Surgery, New York Presbyterian-Columbia University Irving Medical Center, New York, NY; b Division of Cardiology, Columbia University Irving Medical Center, New York, NY

**Figure:**
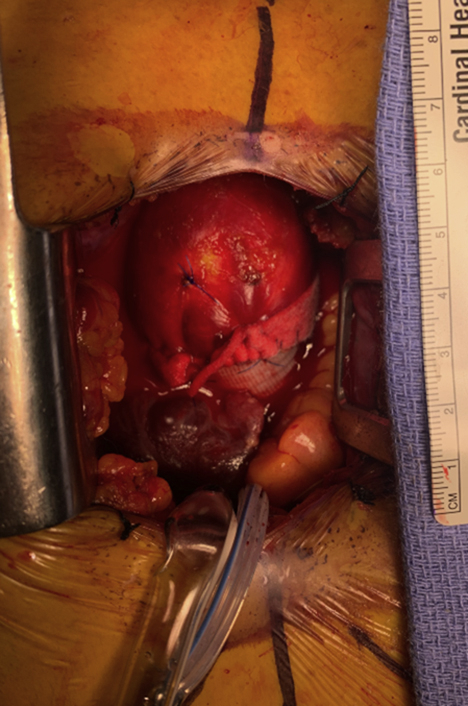
Ascending replacement, in which mildly dilated distal ascending aorta was left behind.

**Central Message**

Although hemiarch repair adds little incremental surgical risk, data do not support its necessity in preventing aneurysmal dilation of the aortic arch.

See Commentary on page 44.

**Feature Editor Note**—Should prophylactic hemiarch resection be performed in patients with bicuspid aortic valve (BAV) disease undergoing proximal aortic surgery? The accompanying article in the *Journal* from Dr Takayama and his colleagues explores the existing evidence. What emerges is that BAV disease is heterogeneous, with different aortic phenotypes, and that the decision to extend a proximal aortic repair into the arch needs to be individualized. Importantly, when followed longitudinally both before and after proximal aortic resection, most BAV aortic phenotypes do not develop aneurysmal disease of the arch. The challenge, therefore, is identifying the minority of patients who are likely to develop proximal arch disease in the future, as these are the ones who may benefit from concurrent prophylactic hemiarch resection at the time of proximal repair. Even this conclusion makes the assumption that the risk of prophylactic repair is less than the risk of reintervention in the future. Although it is clear that the current evidence is limited in scope, current consensus guidelines favor concurrent arch repair for diameters greater than 5 cm (or greater than 4.5 cm in experienced centers).

**Leora B. Balsam, MD**

Bicuspid aortic valve (BAV), a lesion arising from the fusion of 2 aortic valve cusps, is the most common congenital heart defect, with an estimated prevalence of 1.3% worldwide.^1^ The male-to-female ratio is 3:1, with a somewhat-greater reported incidence in white patients.^2^^,^^3^ Although BAV in most cases arises sporadically, in select patients it can present as a manifestation of genetic syndromes such as Turner and Shone complex, as well as in familial inheritance with an autosomal-dominant pattern.^4^, ^5^, ^6^, ^7^ Many patients remain asymptomatic throughout much of their life; in fact, the most common clinical presentation is the incidental discovery of a murmur or early-onset calcification of the aortic valve, and a significant majority of patients have a life expectancy comparable with that of the general population.^2^^,^^3^ Many patients, however, develop a host of cardiac complications that warrant careful monitoring and potential intervention. Aortic valve disease is the most common complication, with clinically significant stenosis (12%-37%) occurring more frequently than regurgitation (13%-32%).^3^ While aortic stenosis tends to occur in older patients compared with regurgitation, it nevertheless manifests earlier than stenosis in patients with normal tricuspid aortic valve (TAV).^3^ Aortic valve replacement is ultimately pursued in >50% of these patients within 25 years.^8^

Aortopathy, which may present as a heterogeneous pattern of aortic dilation in the aortic root, tubular ascending aorta, and aortic arch, is significantly more common in patients with BAV than in the general population. A large, community-based longitudinal cohort study demonstrated that the risk of aneurysm formation within 25 years after BAV diagnosis was 26%—roughly 80 times that of the general population.^8^ One echocardiographic-based retrospective study looking at 280 patients with BAV found a prevalence of ascending aortic dilation to be as high as 56% in patients younger than 30 years of age, which increased to 88% of patients >60 years.^9^ Aortic dissection, the most-dreaded complication of BAV aortopathy, occurs with a frequency 8 times greater than that of the general population.^8^

## Mechanistic Theories and Classification Schema

The potential mechanisms of BAV aortopathy, especially as they relate to the location of aneurysmal dilation, is uncertain, but emerging evidence supports both genetic and hemodynamic driving forces. In support of the hemodynamic theory, Mahadevia and colleagues^10^ demonstrated the presence of asymmetric systolic flow, causing localized variation of wall shear stress along the ascending aorta depending on the specific bicuspid fusion pattern. For example, R-L (right–left) fusion patterns (the most common type) result in an anteriorly directed high-velocity jet and is correlated with aortic root and tubular ascending aneurysms, whereas R-N (right–noncoronary) fusion leads to flow mostly contained in the right-posterior aorta and is associated with distal-ascending and arch aneurysms.^10^^,^^11^ The clinical relevance of these cusp fusion types is unclear, as their presence does not necessarily predict which patients are more likely to sustain unfavorable cardiac events.^8^^,^^12^^,^^13^

Tadros and colleagues^14^ proposed instead a genetically linked pathway whereby reduced levels of fibrillin-1 in patients with BAV compared with their TAV counterparts trigger production of matrix metalloproteinase, which disrupts the integrity of the matrix and promotes vessel dilation. Chim and colleagues^15^ studied the micromechanical and microstructural differences among BAV aneurysms, degenerative aneurysms, and control aortic biopsies. They discovered that BAV aneurysms exhibit at least a 20% greater elastic modulus than the other 2 groups, and the aneurysm groups demonstrate unique localization and distribution of elastin throughout the aortic tissue. In response, McKellar raises^16^ the interesting question of whether point-of-care testing of tissue in the operating room can help guide whether additional aortic resection may be warranted. Other studies supporting a genetic predisposition show that BAV is associated with a larger root and ascending aorta, even in the absence of valve pathology, with a significantly greater growth rate compared with patients with TAV.^17^, ^18^, ^19^, ^20^, ^21^, ^22^ Patients with BAV also have an increased risk of bovine arch,^23^ which itself is associated with aortic aneurysms.^24^ However, the etiologic implications of this association are unclear, as histologic analysis does not yield a conclusive theory on the aneurysmal trigger in bovine arch patients.^25^

Attempts to classify the heterogenous presentations of BAV aortopathy have been made, but no consensus classification system has emerged. One useful categorization scheme that offers prognostic implications in BAV aortopathy draws the distinction between “aortic root phenotype” from that of the “ascending phenotype” first proposed by Della Corte and colleagues.^9^ In the former, BAV aortopathy manifests primarily as aortic root dilation and is associated with aortic regurgitation, younger age of presentation, and more aggressive rates of aneurysmal growth and dissection. In the latter type, aortic dilation develops primarily in the ascending portion, is associated with aortic valve stenosis, later age of presentation, and slower growth rates. Verma and Siu^26^ added to this classification scheme a third group characterized by involvement of the tubular ascending aorta with extension into the transverse aortic arch. Importantly, this group has an association with the R-N fusion type. Subsequently, Fazel and colleagues^27^ proposed a computed tomography–and magnetic resonance imaging–based clustering scheme for aortic dilatation with 4 phenotypes: isolated aortic root dilation (13%), isolated ascending dilation (14%), ascending and arch dilation (28%), and combination aortic root, tubular ascending and transverse arch involvement (45%). Although significant arch involvement is not common in patients with BAV, some groups nevertheless argue for a more-aggressive approach to prophylactic hemiarch procedures in the setting of concomitant proximal aortic surgery, but this remains controversial. Determining the risks of hemiarch extension at the time of proximal aortic replacement weighed against the natural history of the aortic arch in BAV aortopathy after proximal aortic repair is key to resolving this controversy.

## Hemiarch Versus Ascending Aorta Replacement (AAR) for BAV Aortopathy

While numerous consensus guidelines, including the American College of Cardiology/American Heart Association and European Society of Cardiology, offer recommendations for surgical intervention in BAV aortopathy,^28^^,^^29^ these focus on aortic root and ascending aortopathy without addressing the arch. The American Association for Thoracic Surgery does comment that, based on the available literature, arch repair indications in patients with BAV should not differ from those in patients with TAV; namely, if a patient with BAV has an ascending aortic aneurysm with a normal aortic diameter proximal to the innominate takeoff, then it is reasonable to forgo arch intervention. Instead, if the arch diameter is >4.5 cm at the innominate takeoff, hemiarch should be considered, and if the mid-aortic arch diameter is >4.5 cm at the level of the left carotid, total arch replacement can be pursued at experienced centers.^30^ The Canadian Cardiovascular Society does offer specific guidelines with regard to the aortic arch in BAV disease, which includes a class I/B recommendation of aortic arch repair for diameter ≥5.5 cm and a threshold of 5 cm for patients undergoing concomitant cardiac surgery (IIa/C), which may be reduced further to 4.5 cm in experienced aortic centers (IIb/C).^31^

Proper assessment of the risks of prophylactic hemiarch in BAV is a critical consideration to determine the optimal approach for these patients. In particular, the use of adjunctive cerebral protection, such as deep hypothermic circulatory arrest and selective antegrade cerebral perfusion with alternate site arterial cannulation, which is required for hemiarch replacement, increases procedural time, technical complexity, and theoretical risk of neurologic and other end-organ complications as well as bleeding. Greason and colleagues^32^ performed a retrospective study of patients with BAV at their institution comparing survival and intraoperative variables between 225 patients with open hemiarch replacement in one group and 477 patients with clamped ascending aorta replacement in another group. They found that patients in the hemiarch group experienced longer cardiopulmonary bypass (CPB) time (188 vs 97 minutes) and aortic crossclamp time (136 vs 78 minutes) and an increased odds of requiring a blood transfusion (odds ratio, 1.62); however, overall survival and reoperation rates were not significantly different between the 2 groups at 5.4 years' follow-up.^32^^,^^33^ Sultan and colleagues^34^ had similar findings in their study examining 248 patients with hemiarch and 160 patients with ascending aortic replacement, with 47.1% of all patients having BAVs (49.2% hemiarch, 43.8% non-hemiarch). CPB time was longer in the hemiarch group (210 vs 183 minutes), and aortic crossclamp time trended longer in the hemiarch group (152 vs 144 minutes), but this did not reach statistical significance. With the exception of a greater rate of return to the operating room in non-hemiarch patients, there were otherwise no differences in outcomes between the 2 groups regarding postoperative stroke, dialysis, renal insufficiency, or 30-day and 1-year mortality.^34^ Malaisrie and colleagues^35^ reviewed outcomes of 384 patients who underwent a modified Bentall aortic root replacement (ARR), of whom 177 (46%) had additional hemiarch replacement for aortic arch reconstruction. They found again that the hemiarch group had longer CPB and crossclamp times (186 vs 120.5 minutes and 140 vs 104 minutes, respectively). Thirty-day mortality was 3.0% in the hemiarch group and 1.5% in the non-hemiarch group, but this difference was not statistically significant. No significant differences in rates of stroke, reoperation for bleeding, or 5-year survival were found.

Altogether, these data suggest that the addition of hemiarch may not dramatically increase surgical risk. It should be noted, however, that multiple studies have demonstrated an association between surgeon and center volume with improved outcomes of ascending and arch repair among greater-volume surgeons and centers.^30^ One such analysis comparing low-volume centers with high-volume centers in the Commonwealth of Virginia demonstrated significant differences in perioperative mortality associated with aortic arch repair (25.0% vs 4.7%, *P* = .01). Lower-volume centers had significantly greater rates of renal failure, prolonged ventilation, permanent stroke, and length of stay.^36^ This remains a key consideration in assessing the prospect of a move towards “routine” hemiarch in the BAV population.

**Figure 1:**
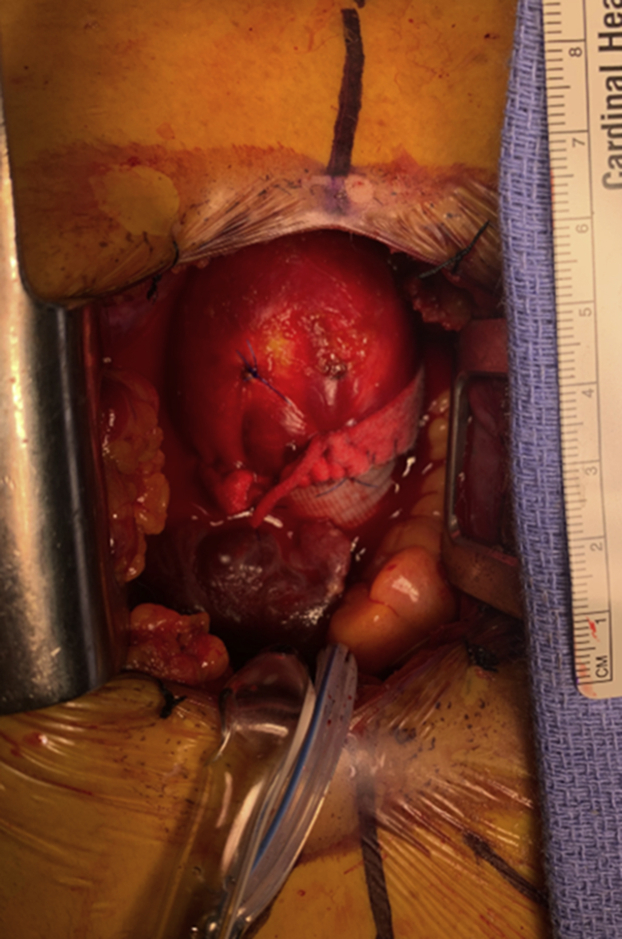
Ascending replacement, in which mildly dilated distal ascending aorta was left behind.

Hemiarch repair may offer technical advantages over isolated ascending replacement.^32^ Clamped ascending aortic replacement carries the risk of leaving behind a significant amount of the ascending aorta to safely apply a crossclamp and perform a secure anastomosis. Indeed, in our experience, postoperative computed tomography scans following ascending aortic replacement often show mild residual ascending aortic dilatation and/or abnormal aortic geometry (Figure 1), although the hemodynamic and clinical significance of these findings is uncertain.

**Figure 2:**
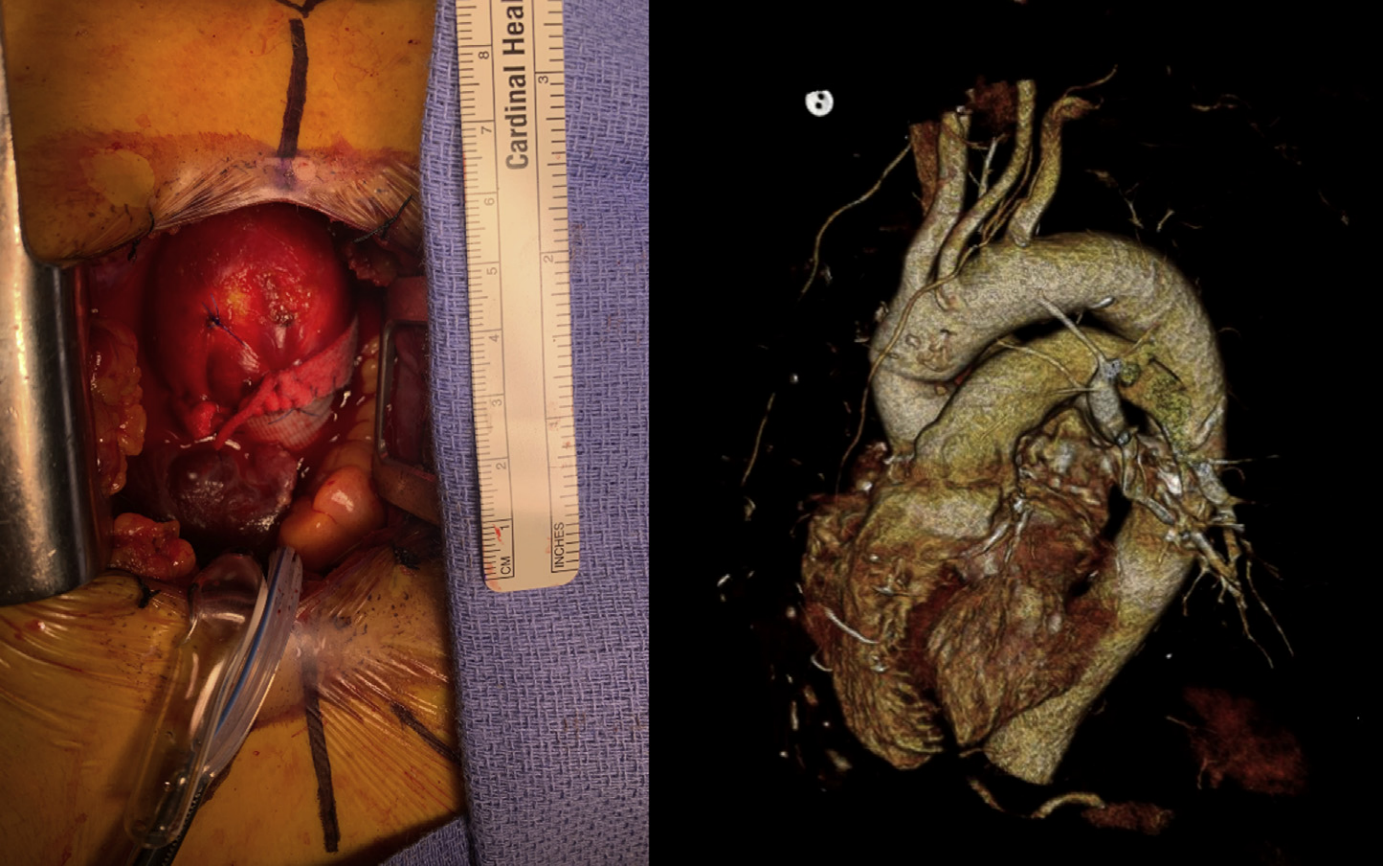
*Left*, Intraoperative picture of minimally invasive aortic root and ascending replacement. The mildly dilated distal ascending aorta was left behind. *Right*, Postoperative computed tomography angiogram showing a “size gap” between the graft and mildly dilated distal ascending aorta after an ascending replacement.

To help determine whether hemiarch is necessary, an important question to answer is whether the residual aortic tissue is of concern for subsequent aneurysmal dilation and adverse events. A number of groups have looked at outcomes of patients undergoing ARR and/or AAR without aortic arch resection, and these results are informative with regard to the behavior of the aortic arch in BAV after a repair of a root/ascending aneurysm. Bilkhu and colleagues^37^ studied 168 patients with BAV undergoing ARR (75.6%) or AAR (24.4%). Median aortic arch dimension was 3 cm (range, 2.4-4.1 cm) preoperatively and remained unchanged at 3 cm (range, 2.4-4.2 cm) at 5.9-year follow-up. This series demonstrated 97% freedom from reoperation, with no patients requiring subsequent surgery on the arch. These findings are in line with a similar study from Iribarne and colleagues,^38^ which showed a rate of reintervention at 9 years' follow-up of only 0.9% among 308 patients with BAV aortopathy undergoing proximal aortic treatment. Park and colleagues^39^ reported no growth and no instances of reoperation of the aortic arch at 4.2 years' follow-up among 422 patients with BAV undergoing AAR or ARR with unresected arch segments. Finally, Abdulkareem and colleagues^40^ followed 395 patients (192 BAV and 203 TAV) who underwent either ARR or AVR and determined that both patients with TAV and BAV exhibited no expansion of the ascending aorta or arch 5 years following AVR. These studies may support the notion that aberrant hemodynamic flow patterns generated by proximal aortopathy is the underlying culprit leading to arch involvement and that this risk is mitigated by proximal aortic repair. These findings are, however, potentially skewed by the exclusion of patients with preoperative arch dilatation who underwent arch replacement and were thus not included for analysis. Furthermore, a small case series with 4-dimensional flow magnetic resonance imaging for wall shear stress assessment suggested that 8 patients with clamped ascending aorta replacement had more residual tissue at-risk compared with 5 hemiarch patients,^41^ although it is uncertain whether the residual tissue would lead to clinically significant pathology. Figure 2 shows an example of “size gap” between the graft and mildly dilated ascending aorta.

Longitudinal data on aforementioned untreated aortic arch in BAV are reassuring that nondilated tissue does not warrant routine aggressive intervention even in the setting of low incremental risk for hemiarch at experienced centers. Stratifying recommendations based on fusion type (ie, taking a more aggressive approach to the arch in patients with R-N fusion) does not yet seem warranted based on the relatively weak data supporting the association between fusion types and clinically relevant dilatation.

Our institution has behaved similarly to peer institutions on this question. For otherwise young and healthy patients with BAV undergoing surgery for proximal aortic aneurysms, it has been our practice to commonly perform hemiarch repair for dimensions >4 cm or when the aortic tissue is macroscopically abnormally thin, as we believe that a hemiarch does not increase overall surgical risk for these healthy patients. For older patients who predominantly present with aortic stenosis and ascending aneurysms, hemiarch replacement is typically withheld in the absence of >4.5 cm arch aneurysms. Our surgical decision-making is individually tailored to each patient, taking into consideration their risk factors. Ultimately, it is imperative to investigate further into this topic to guide best practice.

### Conflict of Interest Statement

The authors reported no conflicts of interest.

The *Journal* policy requires editors and reviewers to disclose conflicts of interest and to decline handling or reviewing manuscripts for which they may have a conflict of interest. The editors and reviewers of this article have no conflicts of interest.
